# The effects of active (hot-seat) versus observer roles during simulation-based training on stress levels and non-technical performance: a randomized trial

**DOI:** 10.1186/s41077-017-0040-7

**Published:** 2017-03-20

**Authors:** Choon Looi Bong, Sumin Lee, Agnes Suah Bwee Ng, John Carson Allen, Evangeline Hua Ling Lim, Arpana Vidyarthi

**Affiliations:** 1 Department of Paediatric Anaesthesia, KK Women’s and Children’s Hospital, 100 Bukit Timah Road, Singapore, 229899 Singapore; 2 Centre for Quantitative Medicine, Office of Clinical Sciences, Duke-NUS Graduate Medical School, 8, College Road, Singapore, 169857 Singapore; 3 Department of Medicine, National University Health System, Singapore, Singapore; 4 Duke-NUS Medical School, 8, College Road, Singapore, 169857 Singapore

**Keywords:** Simulation, Stress, Non-technical performance, Hot-seat, Observers

## Abstract

**Background:**

Active ‘hands-on’ participation in the ‘hot-seat’ during immersive simulation-based training (SBT) induces stress for participants, which is believed to be necessary to improve performance. We hypothesized that observers of SBT can subsequently achieve an equivalent level of non-technical performance as ‘hot-seat’ participants despite experiencing lower stress.

**Methods:**

We randomized 37 anaesthesia trainees into two groups to undergo three consecutive SBT scenarios. Eighteen ‘hot-seat’ trainees actively participated in all three scenarios, and 19 ‘observer’ trainees were directed to observe the first two scenarios and participated in the ‘hot-seat’ only in scenario 3. Salivary cortisol (SC) was measured at four time points during each scenario. Primary endpoint for stress response was the change in SC (ΔSC) from baseline. Performance was measured using the Anaesthetist’s Non-Technical Skills (ANTS) Score.

**Results:**

Mean SC increased in all participants whenever they were in the ‘hot-seat’ role, but not when in the observer role. Hot-seat ΔSC (mcg/dL) for scenarios 1, 2, and 3 were 0.122 (*p* = 0.001), 0.074 (*p* = 0.047), and 0.085 (*p* = 0.023), respectively. Observers ΔSC (mcg/dL) for scenarios 1, 2, and 3 were −0.062 (*p* = 0.091), 0.010 (*p* = 0.780), and 0.144 (*p* = 0.001), respectively. Mean ANTS scores were equivalent between the ‘hot-seat’ (40.0) and ‘observer’ (39.4) groups in scenario 3 (*p* = 0.733).

**Conclusions:**

Observers of SBT achieved an equivalent level of non-technical performance, while experiencing lower stress than trainees repeatedly trained in the ‘hot-seat’. Our findings suggest that directed observers may benefit from immersive SBT even without repeated ‘hands-on’ experience and stress in the hot-seat. The directed observer role may offer a less stressful, practical alternative to the traditional ‘hot-seat’ role, potentially rendering SBT accessible to a wider audience.

**Trial registration:**

ClinicalTrials.gov Identifier NCT02211378, registered August 5, 2014, retrospectively registered.

**Electronic supplementary material:**

The online version of this article (doi:10.1186/s41077-017-0040-7) contains supplementary material, which is available to authorized users.

## Background

In recent years, simulation-based training (SBT) has been widely adopted as an effective modality for teaching non-technical skills, also known as ‘human factors’ [1], particularly in the field of anaesthesia where high-risk events may occur infrequently and trainees rarely have the opportunity to manage these in the clinical setting. SBT using manikins with physical resemblance to real patients and functional task alignment of the simulation environment with appropriate instructional design [2] allows re-creation of these clinical events on demand, replicating substantial aspects of the real clinical environment [3]. This provides anaesthesia trainees with the opportunity for experiential learning and reflection in a structured environment [4]. Non-technical skills, or ‘human factors’, refer to the complex cognitive and interpersonal skills that underlie effective teamwork required to deliver patient care [1] and can be effectively taught through SBT [5, 6]. Repetitive SBT training has been shown to improve non-technical skills performance for learners who actively participated in the simulation scenarios in the ‘hot-seat’ [7].

Traditionally, SBT requires learners to physically participate ‘hands-on’ in the scenario in the ‘hot-seat’ role. These learners in the ‘hot-seat’, actively participating and managing the clinical scenario, experience significant physiological stress [8–10]. How this stress relates to performance in SBT has yet to be fully explored. It is often assumed that these ‘hot-seat’ learners stand to benefit most from the SBT, as their ‘hands-on’ experience represents a crucial component of Kolb’s experiential learning cycle [11] and the stress they experience during SBT is thought to enhance performance and learning. However, whether the stress experienced in the simulated environment actually contributes to improved performance in SBT remains unknown. While moderate stress has indeed been shown to enhance memory [12], excessive stress has also been shown to have a negative impact on attention [13], memory, decision-making, and group performance [14], and ultimately, impairing overall performance [15].

SBT is resource-intensive [16]. Putting every trainee in the ‘hot-seat’ poses significant constraints on faculty time, scheduling, and finances. Having trainees in the same room actively observing their peers in the ‘hot-seat’ may provide a potentially useful learning opportunity in the SBT environment. However, many educators are unsure if such observers will actually benefit by just observing but not actually participating in the scenario. Experiential learning is viewed as fundamental to simulation and clinical practice [17]. Since observers do not get a concrete ‘hands-on’ experience and are not expected to experience the same levels of stress as those in the ‘hot-seat’, it may be assumed that they would have a less optimal learning experience and their subsequent performance may not be as good as those who were trained in the ‘hot-seat’. It is plausible, though, that active, directed observation may be nearly as effective as being in the ‘hot-seat’ in attaining subsequent non-technical performance.

We aimed to explore the differences between stress levels and non-technical performance between ‘hot-seat’ trainees and directed observers in SBT. We randomized anaesthesia trainees undergoing three consecutive sessions of high-fidelity SBT to ‘hot-seat’ or ‘observer’ groups and compared their stress levels and performance using standardized measures. We hypothesized that (i) ‘observers’ experience less stress than ‘hot-seat’ trainees during SBT sessions overall and (ii) ‘observers’ can achieve an equivalent level of non-technical performance as ‘hot-seat’ trainees in the third subsequent scenario despite not experiencing repeated stress of being in the ‘hot-seat’ in two prior SBT scenarios.

## Methods

### Trial design

We conducted a prospective, randomized single-centre study in the simulation centre of KK Women’s and Children’s Hospital, a tertiary paediatric hospital in the Republic of Singapore. (ClinicalTrials.gov Identifier NCT02211378).

### Participants

Following SingHealth Centralized Institutional Review Board approval and informed consent, 51 anaesthesia trainees, aged 26 to 34, in their second or third year of anaesthesia training, who had just completed 2 months of basic training in the paediatric anaesthesia module, were enrolled in the study. Trainees with a history of anxiety, depression, or psychiatric illness; those who recently experienced stressful or traumatic life events (including bereavement, marriage, divorce, or participation in professional examinations); those with a current illness, pregnancy, medical conditions (including cardiovascular, neurological, metabolic, or endocrine conditions); and those who were on beta blockers, calcium channel blockers, or vasoactive drugs, were excluded from the study. Trainees who had previously participated in three or more previous simulation training sessions were also excluded. Trainees did not participate in SBT on the day that they were on call.

### Interventions

Thirty-seven trainees were block-randomized into two groups to undergo three consecutive SBT scenarios; each session scheduled 1 week apart. Randomization was achieved by computer-generated random numbers in sealed opaque envelopes in blocks of six or seven.

#### Simulation-based training

All the simulation training was conducted in the KK Hospital Simulation Center. The simulation environment was created to mirror the operating suite pre-operative patient reception area or the post-anaesthesia care unit. The Laerdal SimBaby™ was used as the manikin for all three scenarios. Trainees had access to all monitoring and equipment as they would in the real operating suite. We created our SBT using established SBT pedagogy [18], based on thoughtful scenario design linked to intended performance outcomes, identification of relevant crisis resource management (CRM) issues during the actual scenario, and facilitated debriefing of the simulated crisis experienced by a skilled facilitator. The objective of the training was to develop cognitive and interpersonal skills that underlie effective teamwork and communication between anaesthesia trainees and anaesthesia nurses during the simulated crises. Particular emphasis was placed on the need for clarity in communication, mutual support, calling for help early, situation awareness, and utilization of resources. The clinical management of the crises was also explicitly discussed during the debriefing process.

#### Scenario design

We developed scenarios depicting paediatric anaesthesia crises that trainees might encounter during their regular clinical practice. Three standardized scenarios were used, one for each training session, scheduled 1 week apart. The first scenario was based on the recognition and management of anaphylactic shock in a 6-month-old infant in the post-anaesthesia care area (PACU). The second scenario was based on the pre-operative assessment of an 8-month-old infant in septic shock due to suspected intra-abdominal sepsis, and the third scenario was that of an infant in the PACU with airway obstruction secondary to a retained throat pack. These scenarios were specifically designed to achieve particular learning objectives and were of similar complexity. Details of the scenarios and their learning objectives are summarized in Table 1. There was no strenuous physical activity involved in any of the scenarios except possibly for a brief period of chest compressions on the infant manikin, not exceeding a total of 1 or 2 min. The scenarios were terminated between 12 and 15 min either after the patient was stabilized for transfer to ICU or after a senior anaesthetist stepped in to assist in management. These scenarios had been previously tested in random order in two previous pilot studies involving 19 anaesthesia trainees in our institution and assessed by the trainees to be of equivalent difficulty levels. In the pilot studies, during any given SBT session, there was no significant variation in non-technical performance scores between trainees regardless of which scenario was used.

**Table 1:** Scenario objectives and roles

Scenario	Synopsis	Learning Objectives	Role	
			Observer group	Hot-seat group
1	6-month-old infant in PACU with anaphylaxis The patient was in PACU following incision and drainage of an abscess and received antibiotics just prior to arrival. The PACU nurses noticed that the infant was tachycardic and in respiratory distress. The trainee was asked to assess the patient. Expected actions: Obtain a pertinent history, perform a physical examination (bilateral rhonchi, HR 160, BP 65/40), and call a senior for help. Institute appropriate management with the team (oxygen, fluids, adrenaline). If treatment is delayed for 10 min, the patient would have a cardiac arrest. Expected actions: Perform resuscitation with the team according to the PALS algorithm. Arrange for ICU admission.	Technical: Recognition and management of anaphylaxis Non-technical: Calling for help early Communication Read back, feedback Situational awareness when patient deteriorates Teamwork and task management during resuscitation	Observer	Hot-seat
2	8-month-old infant in pre-operative area with septic shock The infant was scheduled for diagnostic laparoscopy. He was febrile and had not been feeding well for 2 days. His mother (confederate) was present and very anxious. She became disruptive as the patient started grunting. Expected actions: Obtain a pertinent history from the mother, assess and manage the patient, and delegate a second nurse to attend to the mother. Work with the nurses to administer oxygen, call a senior for help, institute appropriate monitoring, and alert the surgeon. The patient was tachycardic and hypotensive. If no isotonic fluid was administered by 12 min, the patient would deteriorate into cardiac arrest (PEA). Expected actions: Perform resuscitation with the team according to the PALS algorithm.	Technical: Recognition and management of septic shock, appropriate fluids, inotropes Non-technical: History-taking Appropriate management of caregiver Calling for help early Effective communication with anaesthesia nurses, surgeon, and team	Observer	Hot-seat
3	9-month-old infant in PACU with airway obstruction The infant had just undergone a cleft palate repair and was extubated awake. The PACU nurse noticed that the infant had ‘noisy breathing’ and called the trainee to assess him. The infant was initially coughing but subsequently developed stridor, suprasternal retractions. SpO_2_ 90%, decreasing steadily. Expected actions: Recognize upper airway obstruction and institute appropriate management (exclude foreign body, suction, oxygen, CPAP). Call a senior for help and review the anaesthesia chart. Attempt direct laryngoscopy. Sedation and succinylcholine was needed as the infant coughed on attempted laryngoscopy. The i.v. cannula was found to have extravasated, and a decision had to be made whether to attempt resinsertion of the i.v., give i.m. succinylcholine, or call for more help. Direct laryngoscopy would reveal the retained throat pack, but the patient would develop a brief period of profound hypoxia and bradycardia (HR 30) which would be reversed by oxygenation and a brief period of chest compressions (30 s). Expected actions: Ensure adequate oxygenation and institute brief period of chest compressions if necessary. Communicate with senior anaesthetist and surgeon regarding the retained throat pack and arrange for post-operative admission.	Technical: Recognition of airway obstruction in an infant Differential diagnoses and management. Non-technical: Calling for help early Decision-making: identifying options, balancing risks, reevaluating Situational awareness when patient develops profound bradycardia Communication with nurses and surgeon Effective resource management and teamwork during resuscitation	Hot-seat	Hot-seat

#### Protocol

**Fig. 1:**
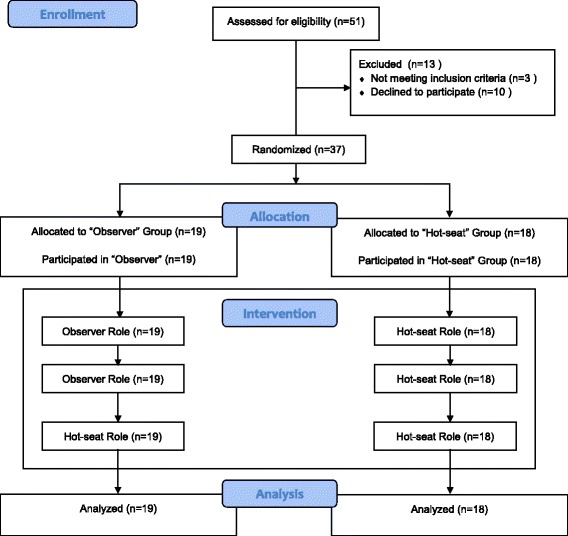
CONSORT flow diagram

Thirty-seven trainees were randomized to either the ‘hot-seat’ group or the ‘observer group’ (Fig. 1).

#### Hot-seat group

The ‘hot-seat’ group consisted of 18 trainees who were in the ‘hot-seat’ role for all three scenarios in which they were the primary anaesthetist actively managing the crisis with two anaesthesia nurses. These ‘hot-seat’ trainees did not participate as observers in any other SBT scenario.

#### Observer group

Trainees in the ‘observer’ group underwent the same pre-briefing outlining the objectives of the SBT and were oriented to the SBT environment and manikin together with the ‘hot-seat’ trainees. They were directed to actively observe the ‘hot-seat’ trainees during the first two SBT scenarios. They were seated against the wall in the same room where the scenario was taking place and instructed before the start of the scenario to remain silent and ‘invisible’ and to observe attentively as they were expected to share their observations during debriefing. They were informed that they might be required to assist but were actually not called on to assist in any of the first two scenarios. During the third scenario, the ‘observer’ trainees assumed the ‘hot-seat’ role as the primary anaesthetist actively managing the crisis with anaesthetic nurses. For every scenario, there was one trainee in the ‘hot-seat’ and two or three ‘observers’. Each trainee in the ‘observer’ group only observed a total of two scenarios (scenarios 1 and 2). When trainees in the ‘observer’ group assumed the ‘hot-seat’ during the third scenario, they too had observers, who were other anaesthesia trainees not involved in the study. The nurses were instructed that the scenarios were designed primarily for the anaesthesia trainee to learn non-technical skills and they were to perform their roles but not be overly ‘helpful’ or to take over the management of the scenario.

To account for the diurnal variation in salivary cortisol, each session was conducted from 2 to 5 p.m. in the afternoon. All the sessions were conducted by the same facilitator and comprised of three phases: pre-briefing, scenario, and debriefing.

##### Pre-briefing

All trainees (including observers) and anaesthesia nurses participated in the pre-briefing. The first pre-briefing session consisted of an introduction to the aims and expectations of the SBT session. Trainees were assured that they were in a safe learning environment and that their SBT performance would be confidential. This was followed by a 15-min mini-lecture on the principles of crisis resource management (CRM) including role clarity, teamwork, communication, personnel support, management of resources, and global assessment. These principles were then illustrated by a group activity (tennis ball game) designed to demonstrate that with increasing chaos, there was a need for role clarity, effective communication, and application of several CRM principles. This was then followed by an orientation to the simulation room environment, equipment, and manikin capabilities. The second and third SBT sessions were similar to the first session, except that the pre-briefing phase started with orientation to the manikin without the introductory mini-lecture and tennis ball game.

##### Orientation

After the pre-briefing, all participants were oriented to the simulation environment and the Laerdal SimBaby™ manikin. Depending on the scenario, the simulation environment was designed to look like the pre-operative or recovery area of the operating room. The equipment and monitors are similar to those in the actual clinical area, and participants were shown where to find the drugs, airway equipment, and defibrillator. They were also instructed to use the telephone to communicate with other personnel if required. They were introduced to the manikin’s capabilities such as its ability to mimic stridor, wheeze, and cough and instructed on how to feel the brachial pulse and auscultate the heart and breath sounds. This session typically lasted approximately 10 min.

##### Scenario

The trainees then underwent the simulation scenario lasting approximately 12–15 min (Table 1, scenario and roles).

##### Debriefing

Immediately after the scenario, all participants (‘hot-seat’ and ‘observer’ trainees as well as nurses) participated in a video-facilitated debriefing conducted by a trained facilitator, lasting approximately 20–25 min. The trainees were assured that they were in a safe environment in which to discuss mistakes, whether real or perceived. Debriefing typically consisted of three phases: a brief ‘reactions’ phase where the trainee and team’s reactions and emotions were addressed, followed by an ‘understanding’ phase during which the team reviews the diagnosis and thought processes during management of the crisis, and finally, a ‘summary’ phase whereby the team summarized their experience and considered how they might generalize their SBT experience to real life. During debriefing, emphasis was placed on non-technical skills performance, unless there was an obvious gap in technical performance that needed to be addressed. Observers also actively participated in the debriefing process and were encouraged to share their thoughts and observations after the ‘reactions’ phase.

### Outcome measures

#### Objective stress response

**Fig. 2:**
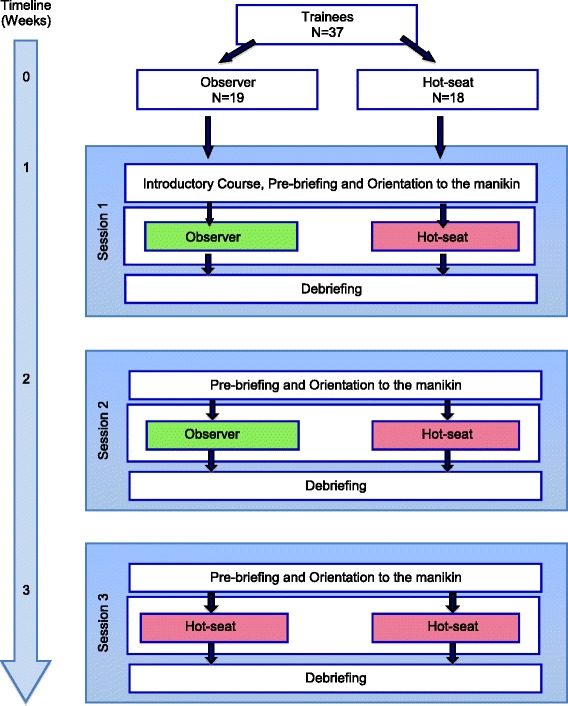
Randomization schedule and study overview. Randomization schedule and time points for outcome measurement

Each trainee’s stress response was measured objectively using heart rate (HR) and salivary cortisol (SC) at four different time points: T0, at baseline prior to orientation; T1, immediately prior to the scenario; T2, immediately after the scenario; and T3, immediately after debriefing (Fig. 2). SC is a well-established biomarker of stress and has been used in numerous clinical and behavioural studies over the past few decades. Cortisol is secreted in response to the activation of the hypothalamic-pituitary-adrenal axis. SC has been shown to be synchronous with serum cortisol concentration across the 24-h time frame [19] and easily measured with a simple enzyme immunoassay [20].

#### Subjective stress response

The subjects’ subjective stress response at the end of debriefing after each simulation session was evaluated using a subjective stress questionnaire, modified from the Depression Anxiety Stress Scale (DASS) [21], to exclude questions relating to depression and include only questions relating to anxiety or stress experienced during the SBT session, thereafter referred to as the modified DASS (mDASS). The mDASS scoring is shown in Additional file 1. The minimum possible score was 0, and the maximum possible score was 72.

#### Data collection

A research assistant not involved in the conduct of the SBT collected all the data. HR was recorded using pulse oximetry (Nellcor). SC samples were collected using Salimetrics oral swabs (Salimetrics) placed under the tongue for 1–2 min, placed in storage tubes and kept frozen at −80 °C. Samples were centrifuged and analyzed in the Salimetrics laboratory (Salimetrics LLC, State College, PA) using the ELISA technique.

#### Performance

In order to maintain confidentiality for the trainees and ensure that their SBT performance would not inadvertently bias their assessment during their paediatric anaesthesia training, we only formally evaluated the trainees’ SBT performance after all participants had completed both the SBT scenarios and their clerkship at our institution. SBT performance was assessed by two independent experts, blinded to trainee group allocation, who reviewed video recordings of the scenarios in random order. The two assessors rated each trainee’s non-technical performance using the Anaesthetist’s Non-Technical Skills (ANTS) score, a previously validated and reliable marking system [22]. This score consists of four categories encompassing task management, team-working, situational awareness, and decision-making. Task management contains specific elements including planning and preparing, prioritizing, providing and maintaining standards, and identifying and utilizing resources; team-working elements include coordinating activities with team members, exchanging information, using authority and assertiveness, assessing capabilities, and supporting others; situational awareness elements include gathering information, recognizing and understanding, as well as anticipating; decision-making elements include identifying options, balancing risks and selecting options, as well as reevaluating. Each specific element is rated from poor (1) to good (4). The maximum possible score was 60. The ANTS scoring and expected performance outcomes are detailed in Additional file 2. The assessors were previously trained in the use of the ANTS score while involved in two pilot studies (*n* = 19) at our institution. For this study, assessors were re-trained in assessing the ANTS score for the three scenarios and their specific objectives over 3 days, a total of 9 h. Assessors calibrated their scores by analyzing the first three videos together. They subsequently rated each trainee’s performance independently. A consensus process resolved inter-rater scoring differences of more than 4 points that involved re-analyzing the video and reviewing the objectives of the scenario. The final ANTS score was the mean of the two ANTS scores rated by the assessors.

### Statistical analysis

#### Sample size calculation

An a priori power calculation based on a two-sample *t* test and using pilot data from 19 subjects indicated that a sample size of *n* = 18 per group would provide 80% power at *α* = 0.05 to detect a clinically meaningful 6-point difference in mean ANTS scores between the ‘hot-seat’ and ‘observer’ groups.

#### Analysis of outcome stress variables

Baseline demographics and participant characteristics were summarized and compared between the ‘hot-seat’ and ‘observer’ groups using a two-sample *t* test for continuous variables and Fisher’s exact test for categorical variables.

**Fig. 3:**
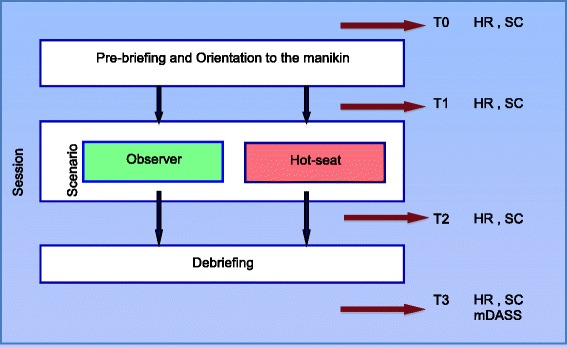
Time points for outcome measurement

Objective stress indicators SC and HR were measured at four time points (T0, T1, T2, T3) during each SBT scenario (Fig. 3). T0 was the baseline when trainees enter the simulation centre, prior to pre-briefing. T1 was just prior to the start of the simulation scenario, T2 was at the end of the scenario, and T3 was at the end of debriefing. We measured the stress indicators based on the results of a previous study [8] which indicated the time course of changes in stress levels during the various phases of the SBT session. The change from T0 (baseline) to T2—represented here as ΔSC and ΔHR in each session—was the most relevant and informative time interval representing the stress response to the scenario.

All four study endpoints (SC, HR, mDASS, and ANTS) were analyzed using a mixed model repeated measures analysis of variance approach with participants modeled as random effects, role (‘hot-seat’ versus ‘observer’) as a fixed effect, and session as a repeated measures fixed effect and normally distributed errors. The variance-covariance matrix was unstructured and blocked on role. Model parameters were obtained using restricted maximum likelihood estimation. Hypotheses of specific interest were tested using contrasts within the repeated measures mixed model construct. Hypotheses of interest involved both within-subject (contrasts among sessions) and between-subject comparisons (contrasts between roles). In the ‘hot-seat’ group only, a test for linear trend over sessions 1–3 was performed on SC, HR, and mDASS to possibly detect evidence of diminishing stress with accumulated stress exposure. In the analysis of SC, we defined a contrast comparing ‘hot-seat’ session 1 with ‘observer’ session 3, as this represents the first hot-seat session for both. The Kenward-Roger approach was specified in the analysis to obtain appropriate denominator degrees of freedom for each hypothesis test and contrast, and to adjust standard errors involving fixed effects. We compared ΔSC and ΔHR among the three sessions by ‘hot-seat’ and ‘observer’ roles. Ninety-five percent confidence intervals were calculated for all estimates.

A linearized cumulative normal plot of the model residuals was used to visually assess adherence to a normal distribution, along with the Kolmogorov-Smirnov (K-S) test. If any departure from normality was found, a non-parametric analysis was also carried out in which session medians were compared within the respective ‘hot-seat’ and ‘observer’ groups using Friedman’s test. Comparisons of hot-seat versus observer medians for each session were performed using the Kruskal-Wallis test.

ANTS scores were analyzed using the repeated measures mixed model. To investigate whether ‘observers’ could achieve a level of non-technical performance equivalent to ‘hot-seat’ trainees in the third ‘hot-seat’ scenario, a contrast was used to compare mean ANTS scores between the ‘observer’ and ‘hot-seat’ groups in session 3, and a 95% confidence interval was obtained on the difference. A difference between ‘hot-seat’ and ‘observer’ roles of 6 points or less in mean ANTS scores was considered clinically equivalent. ICC was calculated on rater agreement between ANTS scores.

Statistical significance was set at *p* ≤ 0.05. We did not adjust for multiple comparisons. In the event of a significant *F* test, *p* values are reported for post hoc contrasts. All analyses were performed using the SAS statistical package version 9.4 (SAS Inc., Cary, NC, USA).

## Results

There were no statistically significant demographic differences or differences in prior clinical and simulation experience between the ‘hot-seat’ and ‘observer’ groups.

### Stress response

#### ‘Hot-seat’ group

**Fig. 4:**
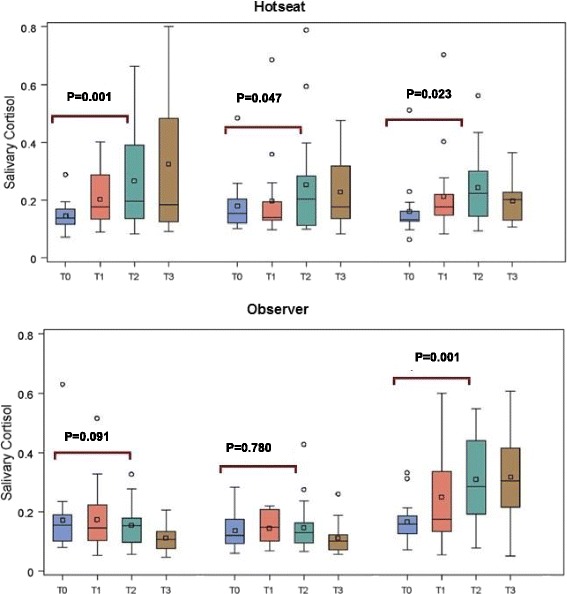
Salivary cortisol change in hot-seat versus observer groups during three SBT sessions. For hot-seat: SC was significantly elevated from baseline in all 3 sessions (t-tests, DF=51), with mean ΔSC (μg/dL) for sessions 1, 2 and 3 of 0.122 (t=3.39, p=0.001), 0.074 (t=2.04, p=0.047) and 0.085 (t=2.35, p=0.023), respectively

Mean SC was significantly elevated from baseline in all three sessions, with mean ΔSC (μg/dL) for sessions 1, 2, and 3 of 0.122 (*p* = 0.001), 0.074 (*p* = 0.047), and 0.085 (*p* = 0.023), respectively. Median SC was significantly elevated from baseline in sessions 1 and 3, with median ΔSC (μg/dL) for sessions 1, 2, and 3 of 0.05 (*p* = 0.020), 0.03 (*p* = 0.065), and 0.05 (*p* = 0.003), respectively (Table 2 and Fig. 4). Mean ΔSC did not differ significantly among the three sessions (*p* = 0.608), and a linear trend test was not significant (*p* = 0.464). In non-parametric analysis, median ΔSC values for sessions 1 to 3 were 0.052, 0.031, and 0.051, respectively, with no significant differences (*p* = 0.311). HR (beats/min) was significantly elevated in sessions 1 and 2, where mean ΔHR were 6.39 (*p* = 0.017), 5.44 (*p* = 0.041), and 4.72 (*p* = 0.075) for sessions 1, 2, and 3, respectively. ΔHR did not differ significantly among sessions (*p* = 0.902), and there was no evidence of a linear trend (*p* = 0.652). Mean subjective stress scores across the three sessions were 17.6, 15.4, and 14.4 and did not differ significantly (*p* = 0.676), again, with no evidence of diminishing stress (*p* = 0.389) (Table 2). For non-parametric analysis, median values for sessions 1 to 3 were 16, 13, and 14.5, respectively, and did not differ significantly (*p* = 0.666).

**Table 2:** Analysis summary on stress and performance variables comparing ‘hot-seat’ (*n* = 18) and ‘observer’ (*n* = 19) study groups

Stress variable	Role	Session			ANOVA *p* values^a^
		1	2	3	
LS mean change from baseline, (95% CI), median					
Salivary cortisol (μg/dL)	Hot-seat	0.12*^b^ (0.05, 0.19) 0.05	0.07* (0.001, 0.15) 0.03	0.09* (0.01, 0.16) 0.05	0.608 0.311^e^
	Observers	−0.06 (−0.13, 0.01) −0.02	0.01 (−0.06, 0.08) −0.01	0.14** (0.07, 0.22) 0.15	<0.001**^d^ 0.008**^e^
	Difference	0.18**^c^ (0.08, 0.29)	0.06 (−0.04, 0.16)	−0.06 (−0.16, 0.04)	
	H-L location shift (95% CI)	0.09** (0.02, 0.26)	0.04 (−0.01, 0.11)	−0.06 (−0.16, 0.04)	
LS mean change from baseline, (95% CI)					
Heart rate (beats/min)	Hot-seat	6.4* (1.2, 12)	5.4* (0.2, 10.7)	4.7 (−0.5, 9.9)	0.902
	Observers	−0.4 (−4.7, 3.8)	−0.4 (−4.7, 3.8)	5.7* (1.4, 9.9)	0.071
	Difference	6.8* (0.2, 13.5)	5.9 (−0.8, 12.5)	−1.0 (−7.6, 5.7)	
LS mean, (95% CI), median					
mDASS	Hot-seat	17.6 (12.4, 22.7) 16	15.4 (10.2, 20.5) 13	14.4 (9.2, 19.6) 14.5	0.676 0.666^e^
	Observers	9.0 (4.9, 13.1) 6	5.9 (1.9, 10.0) 5	13.4 (9.3, 17.4) 11	0.042*^f^ 0.001**^e^
	Difference	8.6* (2.0, 15.1)	9.4** (2.9, 16.0)	1.0 (−5.5, 7.5)	
	H-L location shift (95% CI)	8.0* (2, 15)	8.0** (2, 15)	1.0 (−5, 9)	
LS mean, (95% CI)					
ANTS	Hot-seat	36.7 (34.6, 38.9)	39.6 (37.5, 41.7)	40.0 (37.9, 42.1)	0.036*
	Observers			39.4 (37.4, 41.5)	
	Difference			0.6 (−2.4, 3.5)	

*LS* least squares, *H-L* Hodges-Lehmann

Statistically significant, *p < 0.05; **p < 0.01

^a^H_0_: no difference among session means

^b^H_0_: mean change from baseline = 0 (salivary cortisol and heart rate only)

^c^H_0_: no difference between ‘hot-seat’ and ‘observer’ session means

^d^Post hoc comparisons: session 1 versus 3, −0.20 (−0.31, −0.10); *p* = 0.0002; session 2 versus 3: −0.13 (−0.24, −0.03); *p* = 0.011

^e^H_0_: no difference among session medians

^f^Post hoc comparison: session 2 versus 3, −7.4 (−13.2, −1.7); *p* = 0.012

#### ‘Observer’ group

Trainees in the observer group did not exhibit significant changes in SC during SBT sessions 1 and 2 as observers but did exhibit a significant increase in session 3 when they were in the ‘hot-seat’ as active participants (*p* < 0.001). Mean ΔSC values (μg/dL) for sessions 1, 2, and 3 were −0.062 (*p* = 0.091), 0.010 (*p* = 0.780), and 0.144 (*p* < 0.001), respectively, with significant elevation in session 3 (Table 2 and Fig. 3). Mean ΔSC differed significantly among sessions (*p* = 0.0007) with mean ΔSC significantly higher in session 3 than in sessions 1 (*p* = 0.0002) and 2 (*p* = 0.011) but with no significant difference between sessions 1 and 2 (*p* = 0.162). A similar outcome was observed in a non-parametric comparison of ΔSC for sessions 1, 2, and 3 with median values −0.020, −0.009, and 0.145, respectively. Mean ΔHR (beats/min) for sessions 1, 2, and 3 were −0.42 (*p* = 0.843), −0.42 (*p* = 0.843), and 5.68 (*p* = 0.010), respectively. Mean ΔHR did not differ significantly among sessions (*p* = 0.071). Mean subjective stress scores across the three sessions were 9.0, 6.0, and 13.4, respectively, and differed significantly (*p* = 0.042). Post hoc tests showed a significant increase in subjective stress from session 2 to session 3 (*p* = 0.013) (Table 2).

#### ‘Hot-seat’ versus ‘observer’ group

Differences in mean ΔSC between roles (‘hot-seat’ minus ‘observer’) for sessions 1, 2, and 3 were, respectively, 0.18 (*p* = 0.0005), 0.06 (*p* = 0.216), and −0.06 (*p* = 0.250). A similar pattern was reflected in the non-parametric analysis where the Hodges-Lehmann (H-L) location shifts between the ‘hot-seat’ and ‘observer’ groups in ΔSC for sessions 1–3 were 0.090 (*p* = 0.009), 0.040 (*p* = 0.121), and −0.057 (*p* = 0.289). There was no statistically significant difference in mean ΔSC between ‘hot-seat’ in session 1 and ‘observer’ in session 3. The ΔSC in ‘hot-seat’ and ‘observer’ groups during the three successive SBT sessions are shown in Fig. 3. For ΔHR, session means were 6.8 (*p* = 0.045), 5.9 (*p* = 0.083), and −1.0 (*p* = 0.774), respectively. Differences in mean subjective stress scores were 8.6 (*p* = 0.011), 9.4 (*p* = 0.005), and 1.0 (*p* = 0.757) with these same results reflected in the non-parametric H-L location shifts and significance tests (Table 2).

### Performance

ANTS scores in the ‘hot-seat’ group for sessions 1, 2, and 3 were 36.8, 39.6, and 40.0, respectively, with significant differences indicated by the omnibus *F* test (*p* = 0.036). In addition, the test for a linear trend was significant (*p* = 0.019) reflecting an increase in mean ANTS score over the three sessions. The mean difference (95% CI) in ANTS scores for session 3 between ‘hot-seat’ and ‘observer’ groups was 0.6 (−2.4, 3.5) and did not differ significantly from 0 (*p* = 0.733). In addition, at the 95% confidence level, we can infer that the true difference is less than the targeted 6-point, clinically meaningful difference. Correlation analysis (Pearson, Spearman) showed no evidence of relationship between stress response and performance during any of the sessions, neither in the ‘hot-seat’ group nor in the ‘observer’ group. ICC on performance between raters was ICC = 0.66.

## Discussion

In this randomized study of anaesthesia trainees undergoing SBT, we found that the observer role is less stressful than the ‘hot-seat’ role. The ‘hot-seat’ role is consistently associated with a measurable physiological stress response and greater subjective stress regardless of whether trainees have previously been directed to observe or have repeatedly participated in the ‘hot-seat’. We also found that directed observers of SBT, despite not having ‘hands-on experience’ or experiencing physiological stress responses during the first two prior SBT scenarios, achieved an equivalent level of non-technical performance as those trained in the ‘hot-seat’ during the third scenario. Simulation has been shown to be a stressful experience for learners [23–25]. The stress experienced by the trainees during SBT may result not only from the scenario itself but also from the simulation environment, including emotions generated from the scenario, team dynamics, the presence of observers, and the perception of being appraised.

Several studies in simulation have indeed demonstrated that the stress experienced by the ‘hot-seat’ participants during SBT often exceeds the stress experienced during normal clinical encounters in the ‘real world’ [24, 25]. We found that the trainees in the ‘hot-seat’ group not only reported feeling more stressed, they also experienced a significantly higher level of physiological stress, measurable by increases in heart rate and salivary cortisol, compared to trainees who were observers in the same scenarios. This stress response induced by being in the ‘hot-seat’ did not diminish with repeated exposure to SBT over three consecutive weeks, but this prior experience of stress and active participation in SBT appeared to be associated with a small but statistically significant improvement performance over the course of the three sessions. We did not find any correlation between stress response during SBT and non-technical performance during any of the sessions.

Trainees who were directed to actively observe the first two scenarios did not experience an objective physiological stress response despite being physically present in the same room. They were told that they might be called upon to assist, so they paid attention to details of the scenario. Moreover, the observers actively participated in each debriefing session, sharing their thoughts and observation. Debriefing is perhaps the most important phase of simulation training, and having the opportunity to reflect and discuss the scenarios and other trainees’ performance may be as powerful as being in the scenario itself. In a systematic review of observer roles that optimize learning in healthcare simulation education, O’Regan et al. [26] reported that learning and satisfaction in observer roles is closely associated with observer tools, learner engagement, role clarity, and contribution to the debriefing. Although our observers were not given observer tools, they were provided with role clarity during pre-briefing and directed to the learning objectives of the scenario and they also actively participated in the debriefing. This may explain why the observers’ non-technical performance during the third session was equivalent to the trainees who previously trained in the ‘hot-seat’.

This finding is supported by Bandura’s social learning theory, which posits that people learn from one another, via observation, imitation, and modeling. Bandura proposed that virtually all learning acquired experientially could also be acquired on ‘a vicarious basis through observation of other people’s behaviour and its consequences for them’ [26, 27]. Through observation, learners can build behaviours through watching others’ experience and emotions without having to experience it for themselves. He described four requirements for learning: attention, retention, reproduction, and motivation [27]. In our study, pre-briefing and directed observation provided attention and learner engagement, debriefing provided the opportunity for reflection and retention, the ‘hot-seat’ experience in session 3 provided the opportunity for reproduction of behaviour, and expectation to participate in the impending debriefing may have provided the motivation. All these elements may have contributed to the observers’ equivalent performance in session 3.

Our study showed that stress experienced by hot-seat trainees did not diminish over three SBT sessions. This appears to be in contrast to the theory of stress inoculation training, a form of cognitive behavioural therapy designed to help reduce performance anxiety [28]. Stress inoculation training was developed primarily as a clinical intervention and retains a strong emphasis on individualized training and the intensive involvement of a skilled facilitator. In our SBT, other than creating a ‘safe environment’ for trainees during pre-briefing and debriefing, no deliberate attempts were made to help trainees manage stress and anxiety. Stress inoculation therapy was shown to be effective in reducing performance anxiety with a mean length of training of approximately 6–7 sessions [29]. Our trainees underwent three SBT sessions scheduled 1 week apart; so, even if they had inadvertently received some form of stress inoculation training, the effects may not have been evident over this short period.

Medical trainees and residents are exposed to various stressors daily, including having to treat ill patients, perform high-intensity procedures, pass licensing examinations, and manage their clinical, educational, and social responsibilities. Acute stress is known to have negative effects on health and well-being of medical trainees [30, 31]. In addition, elevated stress levels can impede performance on tasks that require divided attention, working memory, retrieval of information from memory, and decision-making [15]. Our study shows that it is possible for trainees to achieve an equivalent level of non-technical performance during a third SBT session through directed observation of their peers in the ‘hot-seat’ during two prior SBT sessions and actively participating in debriefing. If we are able to optimize the directed observer role in SBT, we can potentially reduce the number of times trainees need to be in the ‘hot-seat’, possibly making SBT a less stressful experience for the trainees without necessarily compromising their performance in non-technical skills. The directed observer role in SBT provides a useful learning opportunity and may possibly enhance the efficiency of training as well as improving cost-effectiveness and the feasibility of SBT in some simulation centres and educational institutions.

There are several limitations to our study. First, we do not know the ‘observers’ non-technical performance at baseline and whether they could have achieved the same level of performance as the third SBT session without even having to observe the first two sessions. However, all the trainees were at the same stage of anaesthesia training and had similar simulation training experience and similar clinical performance rating at baseline; study randomization would have ensured that the baseline non-technical performance of the two groups did not differ significantly. Second, since the SBT scenarios were standardized, trainees could have informed other trainees of the scenarios, leading to improved performance in trainees recruited later in the study. However, this was unlikely, as our analysis showed that the performance of trainees who participated later in the study was no different compared to those who participated earlier in the study. Third, although we demonstrated statistically significant increases in stress markers from baseline whenever the trainees were in the ‘hot-seat’ role but not when they were in the observer role, none of these values exceeded physiologically to normal range [32]. As we do not know what level of stress would constitute clinical significance for each individual, we cannot conclude that these statistically significant increases in stress represent clinically significant stress. It is also possible that some of the stress in the ‘hot-seat’ experience may have resulted from the presence of the observers. However, previous studies have shown that the ‘hot-seat’ experience is stressful even in the absence of observers in the room [8, 23, 24]. Forth, we used a subjective stress score modified from the DASS score [21] to measure subjective stress in the trainees. The DASS tool was originally designed to measure depression, anxiety, and stress and does not account for differences in state anxiety or trait anxiety. We are unsure on how our modification of the tool and removal of the measures of depression will alter the psychometric properties of the tool. Lastly, we only studied one method of observing during SBT, where active observers watched the scenarios as they unfolded and then participated actively in the debriefing. Thus, our findings cannot be extrapolated to situations where trainees are passively observing, for example, via video link in a separate room, or to the real clinical environment.

## Conclusions

In conclusion, our study showed that the observer role is less stressful than the ‘hot-seat’ role but not necessarily less useful. With thoughtful instructional design, learners’ engagement and participation in debriefing, directed observers of SBT, despite not participating ‘hands-on’ or experiencing physiological stress responses during two prior SBT scenarios, can potentially achieve an equivalent level of non-technical performance as those trained in the ‘hot-seat’ subsequently. This allows us to rethink the SBT environment and consider the directed observer role as a pragmatic alternative to the traditional ‘hot-seat’ role. SBT is a powerful tool that has enhanced our ability to teach effectively, and our study shows that the power of the tool has a wider scope than previously thought. While it may appear ideal to train every participant in the ‘hot-seat’ role in SBT, this may not always be possible due to resource limitations. When it is not possible to have every trainee in the ‘hot-seat’ role, the directed observer role may provide an equally valuable learning opportunity in SBT and reduce resource requirements, thus expanding the tool’s reach and making it more accessible to education centres and training programs.

## Additional files

Additional file 1: Subjective Stress Questionnaire (mDASS). (DOC 49 kb)

Additional file 2: DASS Questionnaire. (DOCX 18 kb)

## Abbreviations

ANTS: Anaesthetist’s Non-Technical Skills Score
CRM: Crisis resource management
DDF: Denominator degree of freedom
HR: Heart rate
mDASS: Modified Depression Anxiety Stress Scale
NDF: Numerator degree of freedom
PACU: Post-anaesthetic care unit
SBT: Simulation-based training
SC: Salivary cortisol
