# Supplementary material for: The effects of active (hot-seat) versus observer roles during simulation-based training on stress levels and non-technical performance: a randomized trial
Source: Adv Simul (Lond). 2017 Mar 20;2:7. doi: 10.1186/s41077-017-0040-7 (PMC5806270; doi:10.1186/s41077-017-0040-7)
Supplement: Supplementary file 2 — DASS Questionnaire. (DOCX 18 kb) [file 41077_2017_40_MOESM2_ESM.docx]

| DAS S *Name: Date:* | | |
| --- | --- | --- |
| Please read each statement and circle a number 0, 1, 2 or 3 which indicates how much the statement applied to you *over the past week*. There are no right or wrong answers. Do not spend too much time on any statement. | | |
| *The rating scale is as follows:*  0 Did not apply to me at all  1 Applied to me to some degree, or some of the time  2 Applied to me to a considerable degree, or a good part of time  3 Applied to me very much, or most of the time | | |
| 1 | I found myself getting upset by quite trivial things | 0 1 2 3 |
| 2 | I was aware of dryness of my mouth | 0 1 2 3 |
| 3 | I couldn't seem to experience any positive feeling at all | 0 1 2 3 |
| 4 | I experienced breathing difficulty (eg, excessively rapid breathing, breathlessness in the absence of physical exertion) | 0 1 2 3 |
| 5 | I just couldn't seem to get going | 0 1 2 3 |
| 6 | I tended to over-react to situations | 0 1 2 3 |
| 7 | I had a feeling of shakiness (eg, legs going to give way) | 0 1 2 3 |
| 8 | I found it difficult to relax | 0 1 2 3 |
| 9 | I found myself in situations that made me so anxious I was most relieved when they ended | 0 1 2 3 |
| 10 | I felt that I had nothing to look forward to | 0 1 2 3 |
| 11 | I found myself getting upset rather easily | 0 1 2 3 |
| 12 | I felt that I was using a lot of nervous energy | 0 1 2 3 |
| 13 | I felt sad and depressed | 0 1 2 3 |
| 14 | I found myself getting impatient when I was delayed in any way (eg, lifts, traffic lights, being kept waiting) | 0 1 2 3 |
| 15 | I had a feeling of faintness | 0 1 2 3 |
| 16 | I felt that I had lost interest in just about everything | 0 1 2 3 |
| 17 | I felt I wasn't worth much as a person | 0 1 2 3 |
| 18 | I felt that I was rather touchy | 0 1 2 3 |
| 19 | I perspired noticeably (eg, hands sweaty) in the absence of high temperatures or physical exertion | 0 1 2 3 |
| 20 | I felt scared without any good reason | 0 1 2 3 |
| 21 | I felt that life wasn't worthwhile | 0 1 2 3 |

|  | *Please turn the page ☞* |
| --- | --- |

| *Reminder of rating scale:*  0 Did not apply to me at all  1 Applied to me to some degree, or some of the time  2 Applied to me to a considerable degree, or a good part of time  3 Applied to me very much, or most of the time | | |
| --- | --- | --- |
| 22 | I found it hard to wind down | 0 1 2 3 |
| 23 | I had difficulty in swallowing | 0 1 2 3 |
| 24 | I couldn't seem to get any enjoyment out of the things I did | 0 1 2 3 |
| 25 | I was aware of the action of my heart in the absence of physical exertion (eg, sense of heart rate increase, heart missing a beat) | 0 1 2 3 |
| 26 | I felt down-hearted and blue | 0 1 2 3 |
| 27 | I found that I was very irritable | 0 1 2 3 |
| 28 | I felt I was close to panic | 0 1 2 3 |
| 29 | I found it hard to calm down after something upset me | 0 1 2 3 |
| 30 | I feared that I would be "thrown" by some trivial but unfamiliar task | 0 1 2 3 |
| 31 | I was unable to become enthusiastic about anything | 0 1 2 3 |
| 32 | I found it difficult to tolerate interruptions to what I was doing | 0 1 2 3 |
| 33 | I was in a state of nervous tension | 0 1 2 3 |
| 34 | I felt I was pretty worthless | 0 1 2 3 |
| 35 | I was intolerant of anything that kept me from getting on with what I was doing | 0 1 2 3 |
| 36 | I felt terrified | 0 1 2 3 |
| 37 | I could see nothing in the future to be hopeful about | 0 1 2 3 |
| 38 | I felt that life was meaningless | 0 1 2 3 |
| 39 | I found myself getting agitated | 0 1 2 3 |
| 40 | I was worried about situations in which I might panic and make a fool of myself | 0 1 2 3 |
| 41 | I experienced trembling (eg, in the hands) | 0 1 2 3 |
| 42 | I found it difficult to work up the initiative to do things | 0 1 2 3 |
